# A Practical Treatise on Diseases of the Skin

**Published:** 1853-02

**Authors:** 


*A Practical Treatise on Diseases of the Skin; by* J. Moore Neligan, M. D., F. R. S., Honorary Fellow to the Society of Physicians of Sweden &c. Philadelphia, Blanchard & Lea, 1852, pp. 333.

DISEASES of the skin are no doubt easily diagnosed--by those who have made them an especial study—but we confess that to our uneducated ears the various terms employed by dermatologists, have given rise to much confusion. Between the natural system of Alibert, Wilson, and Cazenave, and the artificial system of Willan, with the modifications by Biett, Cazenave, Schedel, and Bennett, we found ourselves in the condition of the countryman, who, on his first visit to the city, was very much annoyed that the houses were so thick that he could not see the town; we found the names so numerous that it was almost impossible to name the disease.

The work before us is one of the best we have seen on the subject. The classification, which is a modification of that of Willan, is tabulated as follows :—

| ORDER. | GENERA. |
|---|---|
| 1. EXANTHEMATA, - | Erythema, Erysipelas, Urticaria, Roseola. |
| 2. VESICULÆ, - - | Eczema, Herpes, Pemphigus, Rupia, Scabies. |
| 3. PUSTULÆ, - - | Acne, Impetigo, Ecthyma. |
| 4. PAPULÆ, - - | Lichen, Prurigo. |
| 5. SQUAMÆ, - - | Psoriasis, Pityriasis. |
| 6. HYPERTROPHIÆ, - | Ichthyosis, Molluscum, Stearhœa, Elephantiasis, Verruca, Clavus, Callositates, Condylomata, Nævus. |
| 7. HÆMORRHAGIÆ, - | Purpura. |
| 8. MACULÆ, - - | Vitiligo, Ephelis. |
| 9. CANCRODES, - - | Lupus, Kelois. |
| 10. DERMATOPHYTÆ, - | Porrigo, Sycosis. |

*Supplementary Groups.*

SYPHILIDES.

DISEASES OF THE APPENDAGES OF THE SKIN.

The descriptions are plain and definite, and the methods of treatment which have been found most beneficial, briefly indicated. We observe that the credit of having first used successfully collodion in Erysipelas is given to Spengler & Rapp. We believe that Dr. J. W. Freer, of this city, was the first who used it. The French writers have done justice in this matter, but the English can see light only in the direction of the continent.

The closing chapter is devoted to the general points in therapeutics specially applicable to this class of affections. The author places but little confidence in baths; he thinks salt water bathing, although it seems frequently to produce beneficial action, is generally injurious. Ointments, cerates, &c., are discussed and formulæ given. Internal remedies are almost always necessary, and require to be persisted in a long time; arsenic and iodine are in favor with our author, as they are with most practitioners.

For sale by Keen, Bro. & Co. J.

